# Linking ADHD and Behavioral Assessment Through Identification of Shared Diagnostic Task-Based Functional Connections

**DOI:** 10.3389/fphys.2020.583005

**Published:** 2020-12-17

**Authors:** Chris McNorgan, Cary Judson, Dakota Handzlik, John G. Holden

**Affiliations:** ^1^Department of Psychology, University at Buffalo – SUNY, Buffalo, NY, United States; ^2^Department of Computer Science, University at Buffalo – SUNY, Buffalo, NY, United States; ^3^Department of Psychology, University of Cincinnati, Cincinnati, OH, United States

**Keywords:** ADHD, functional networks, fMRI—functional magnetic resonance imaging, machine learning, Iowa Gambling Task

## Abstract

A mixed literature implicates atypical connectivity involving attentional, reward and task inhibition networks in ADHD. The neural mechanisms underlying the utility of behavioral tasks in ADHD diagnosis are likewise underexplored. We hypothesized that a machine-learning classifier may use task-based functional connectivity to compute a joint probability function that identifies connectivity signatures that accurately predict ADHD diagnosis and performance on a clinically-relevant behavioral task, providing an explicit neural mechanism linking behavioral phenotype to diagnosis. We analyzed archival MRI and behavioral data of 80 participants (64 male) who had completed the go/no-go task from the longitudinal follow-up of the Multimodal Treatment Study of ADHD (*MTA 168*) (mean age = 24 years). Cross-mutual information within a functionally-defined mask measured functional connectivity for each task run. Multilayer feedforward classifier models identified the subset of functional connections that predicted clinical diagnosis (ADHD vs. Control) and split-half performance on the Iowa Gambling Task (IGT). A sample of random models trained on functional connectivity profiles predicted validation set clinical diagnosis and IGT performance with 0.91 accuracy and d′ > 2.9, indicating very high sensitivity and specificity. We identified the most diagnostic functional connections between visual and ventral attentional networks and the anterior default mode network. Our results show that task-based functional connectivity is a biomarker of ADHD. Our analytic framework provides a template approach that explicitly ties behavioral assessment measures to both clinical diagnosis, and functional connectivity. This may differentiate otherwise similar diagnoses, and promote more efficacious intervention strategies.

## Introduction

Attention-deficit hyperactivity disorder (ADHD) is the most commonly diagnosed psychological disorder among school-aged children and across the lifespan (Bell, 2010). Moreover, a study of children aged 8–16 years found that 70% of children with a clinical ADHD diagnosis also had some form of learning disability, highlighting the cognitive developmental challenges that often accompany attention disorders (Mayes et al., 2000). A clinical definition of ADHD is complicated by the prevailing view that it spans a continuum (Graham and Madigan, 2016) and exists as multiple subtypes (Garon et al., 2006). As of the fourth edition of the *Diagnostic and Statistical Manual of Mental Disorders* (DSM-IV), functional impairment became mandatory, however, even though the impact of ADHD has been well-studied, its fluid diagnostic criteria remain a challenge in research and clinical settings (Fortes et al., 2020). Moreover, though ADHD is diagnosable cognitive disorder in adults, the developmental stability of the diagnosis is quite poor (Todd et al., 2008).

The present study addresses these challenges with an exploration of brain-network and behavioral differences between a group of young adults with a childhood diagnosis of ADHD and an age-matched group of control participants. We apply a machine learning approach to analyzing these differences with the joint complementary goals of identifying a clinically diagnostic neural connectivity signature of ADHD and relating the underlying neural processing dynamics to performance on a commonly used behavioral diagnostic task. Furthermore, though connectivity is most commonly measured using linear correlations among time series, our use of cross-mutual information based measures of functional connectivity highlights the important role that alternative indices of functional connectivity can play in exploring brain-behavior correlations. By identifying task-related functional connections that are both diagnostic and predictive of clinically relevant task performance, we identify neural pathways that may be implicated in different ADHD subpopulations, and provide a means by which different populations may be behaviorally identified. Together, these results inform how multiple diagnostic tools may be integrated to better distinguish diagnostic subtypes, and evaluate potential interventions.

### The Iowa Gambling Task as a Behavioral Indicator of ADHD

The Iowa Gambling Task (IGT) is a computerized assessment that presents individuals with realistic gambling decisions, and is used experimentally to investigate normal and disordered decision making and adapted for clinical use (Lin et al., 2019), including for clinical diagnosis of ADHD (Toplak et al., 2010). The task assigns the participant an initial imaginary monetary account, and asks them to select cards from one of four decks, causing a gain or loss from this account. Two of the decks are high-variance, and two are low-variance, with respect to potential gains or losses, introducing an element of risk (Bechara et al., 2005).

Evidence for behavioral ADHD-related differences in the IGT among adults and children is mixed; some studies show worse performance for ADHD participants, and others show no difference from controls (Groen et al., 2013). The task implicitness may be an important factor in its diagnostic accuracy for children. The IGT is theoretically motivated by the Somatic Marker Hypothesis (SMH) (Damasio, 1996), which maintains that physiological changes in the body (somatic markers; e.g., sweating palms) are correlated with and interpreted as emotional states. Somatic markers and their evoked emotions are associated with events and decision outcomes, and shape behavior (Damasio, 1994). The utility of the IGT in evaluating ADHD rests on the observation that abnormal emotion processing is associated with impaired decision-making (Bechara et al., 2005). Roshani et al. (2020) found that both significant differences in IGT score and in IGT decision making times discriminates ADHD from controls, with adult ADHD participants less likely to favor advantageous decks and making faster deck selections. This pattern suggests that the task taps ADHD participants’ proneness to making riskier and more impulsive choices related to abnormal emotion processing in ventromedial prefrontal cortex (vmPFC) (Bechara et al., 1994).

### Neural Processing Dynamics as an Indicator of ADHD

In contrast to behavioral results, ADHD and control populations appear to show more reliable neural processing differences in the IGT. Conventional general linear model analyses (GLMA) test group or condition differences in regional blood oxygen level dependent (BOLD) functional magnetic resonance imaging (fMRI) signals, which are indicators of neural activity. Within healthy controls, GLMA studies show the network of brain regions that are recruited by the task appears to dynamically change as the task progresses, and the task history changes participant’s expectations (Lin et al., 2008). When comparing ADHD to healthy controls, GLMA studies typically show that ADHD participants significantly under-activate the left and right precuneus, putamen and caudate when choosing higher-reward decks as compared to controls (Norman et al., 2018). These regions are implicated in the dopaminergic reward system, suggesting that irregular processing within this network may be a factor in the behavioral markers of ADHD. A recent GLMA study by Yang et al. (2019) examined group differences during the IGT in orbitofrontal cortex, a region that is sometimes grouped with the larger vmPFC, and is part of the putative reward network. This study found that adults with ADHD exhibited both lower orbitofrontal cortex activation and poorer performance on the IGT than healthy controls. Thus, though the behavioral literature supporting the clinical utility of the IGT in detecting ADHD is mixed, the neuroimaging literature suggests that the task’s sensitivity hinges on the recruitment of different networks in controls and ADHD populations.

### Neural Circuitry Implicated in Attention Disorders

All networks are described in terms of nodes and the connections between them, but they differ in composition across domains. As appropriate graph-theoretic methods have been developed, cognitive neuroscientists have increasingly employed fMRI to undertake *in vivo* explorations of brain networks. In the neuroscientific domain, nodes in brain-based models of cognitive processes correspond to brain regions, and their connections refer to functional, effective, or anatomical connectivity among the brain regions, though we will primarily focus on functional connectivity—defined as a temporal coherence between activity in two regions (Honey et al., 2009). The literature implicating a role for networks in ADHD assumes that connectivity (of any sort) among brain regions critically determines how regional processing and interactions unfold in ADHD. Consequently, connectivity among and within several identifiable networks has been explored as a potential factor in ADHD (Castellanos and Proal, 2012).

Because it is often characterized as a self-regulation disorder, early investigations of ADHD focused on a dysfunctional frontal-subcortical circuit (Voeller, 2004), which is widely regarded to play a critical role in the regulation of attention and impulsivity (Chow and Cummings, 1999; Bonelli and Cummings, 2007). Additionally, activation in another frontal subnetwork implicated in reward processing correlates with ADHD symptom severity (Stark et al., 2011). This suggests that underactivity within the dopaminergic reward system may also play a role in ADHD symptomology.

Increasingly, ADHD has been viewed as a disorder of the default mode network (DMN), as inhibition of the default brain network is associated with poorer performance on many attention-dependent tasks (Buckner et al., 2008). The DMN is a task-negative (i.e., deactivated during task) network of regions believed to comprise distinct but connected subsystems (Buckner et al., 2008; Andrews-Hanna et al., 2010) that develop into adulthood, becoming increasingly integrated with age (Fair et al., 2008). Because the DMN can be explored using resting-state MRI, a clinical advantage of this paradigm is that it does not require patients to perform cognitively demanding tasks (Bullmore, 2012), which may be especially challenging for children with attentional deficits. That said, a review of studies of functional connectivity as a biomarker of ADHD between 2008 and 2017 found widely variable diagnostic accuracies, ranging from 0.55 to 0.95 (Du et al., 2018). Most of the reviewed studies employed black box classifiers that were applied with the goal of optimizing diagnostic accuracy, rather than uncovering theoretical mechanisms underlying specific functional connections.

Though the DMN is a task-negative network, task-positive activity is associated with increased functional connectivity relating the dorsolateral prefrontal cortex to the DMN (Buckner et al., 2008). Effortful attention during tasks requires a switch from the brain’s default mode to an active mode, and fMRI BOLD analyses indicate a pattern of alternating low-frequency activity between task-positive and task-negative activities (Fransson, 2005). Mind wandering is one of the prototypical characteristics of ADHD, and is argued to be negatively associated with activation of the ventral anterior cingulate cortex (ACC), the precuneus, and the temporoparietal junction—all regions within the DMN (Mason et al., 2007; Christoff et al., 2009). Because DMN activity normally decreases during tasks, Konrad and Eickhoff (2010) suggest that failure to inhibit DMN activity may be a neural signature of ADHD. The authors, however, note that the literature is inconsistent with respect to the causal role of functional connectivity, with different models characterizing ADHD as either hyperconnectivity (Tian et al., 2006) or, conversely as hypoconnectivity (Castellanos and Tannock, 2002) of the DMN.

Much of the work on functional connectivity focuses on resting state MRI (rs-MRI), and therefore on connectivity within the task-negative DMN. As observed by Castellanos and Aoki (2016), one of the challenges of rs-MRI studies is that, in the absence of a model task signal, statistical artifacts related to head motion introduce a confounding source of variability in the signal that is difficult to disentangle from signals of interest; the problem is compounded by the increased proneness of ADHD populations to excessive head movement. The authors argue that these obstacles necessitate development of novel analytic procedures on large open datasets. Moreover, Gonzalez-Castillo and Bandettini (2018) argue that important differences exist between resting-state and task-based functional connectivity, and that the reconfiguration that brain networks undergo during tasks inform the neural bases of cognitive processes. This point is especially relevant to the study of ADHD, given the studies cited earlier showing that the network recruitment under the IGT is dynamically dependent on the progression of the task, suggesting that network dynamics when inhibiting and exhibiting behaviors are important for understanding how those with ADHD perform the task.

The go/no-go task has been widely used in neuroimaging studies of ADHD, because it is assumed to rely heavily on the interaction between attention and response inhibition (Simmonds et al., 2008; Hwang et al., 2019). It has been argued more recently (Michelini et al., 2019) that atypical task-based functional connectivity in individuals with childhood ADHD may persist into adulthood. Taken together, these findings suggest that a neural signature of ADHD may be found within task-based functional connectivity from the go/no-go task, even from young adults, advancing this approach as a potential detector of biomarkers that may address the poor stability of ADHD diagnosis (Guo et al., 2020).

### The Present Study: Identification of a Persistent Task-Based FC Signature of ADHD

The present builds upon previous neuroimaging studies exploring task-dependent connectivity from the go/no-go task to investigate the persistent connectomic signature of childhood ADHD in young adults. We use a series of multilayer feedforward classifier models to predict clinical diagnosis and performance on the IGT and the architecture of these models permit classification of embedded groups, and consequently accommodate otherwise inconsistent relationships. For example functional connection X might be diagnostic of ADHD if Y and Z are also strong, but not diagnostic otherwise. We will show that task-based functional connectivity reliably predicts ADHD diagnosis and IGT performance, and that a small number of the most diagnostic connections permitted nearly equivalent accuracy. Moreover, we will show that the machine learning classifiers can be constrained to take advantage of joint probability distributions to identify the functional connections that predict both ADHD diagnosis and IGT performance, establishing the neural bases for the diagnosticity of the IGT and a potential means of identifying ADHD subtypes on the basis of behavioral test performance.

## Materials and Methods

### Archival Data Set and Participants

We analyzed archival MRI and behavioral data from the longitudinal follow-up of the NIMH-sponsored Multimodal Treatment Study of ADHD (*MTA 168*). The MTA was a multisite study designed to evaluate ADHD treatment strategies, and included nearly 600 children, ages 7–9, who were randomly assigned to one of four treatment modes: medication, behavioral, combination medication and behavioral, or routine community care. Parents heard about the study through health care providers, teachers or advertisements, and contacted the investigators who interviewed the children and parents to determine eligibility. Our dataset included the 80 adult participants (64 male) from the *MTA 168* study who had completed the go/no-go fMRI task. Of these, 55 had received an ADHD diagnosis during childhood, and the remainder were age-matched controls. The mean age of the participant subset at scan time was 23.97 years (*SD* = 1.29). The *MTA 168* study procedures for diagnosis, treatment specifics, and sample demographics have been described elsewhere (MTA Cooperative Group, 1999), and we used the diagnostic and behavioral metadata provided with the data set for model training.

### MTA Design and Procedure

The archival Go/No-Go fMRI task data were generated from the study described in Rasmussen et al. (2016), and the reader should refer to the original study for further details. Briefly, the Go/No-Go task used a randomized jittered event-related design, and required participants to respond via button press when presented with a target image, but withhold response when presented with a non-target image. Echo planar functional images were acquired over 154 volumes using the following acquisition parameters: TR (repetition time; the period required for 1 complete volume acquisition) = 2,000 ms; TE (echo time; the period between an RF pulse and its gradient echo) = 30 ms; 32 axial slices; voxel size = 3.4 × 3.4 × 4.0 mm, Slice Gap = 1 mm. T1-weighted images were acquired using the following parameters: TR = 2170 ms; TE = 5.56 ms; 160 sagittal slices; voxel size = 1 × 1 × 1.2 mm. The *MTA 168* study used the IGT procedure described in Bechara et al. (1994). For the *MTA 168* study, IGT score was calculated by subtracting disadvantageous card choices from the advantageous card choices.

### Functional Data Processing

We applied here the data processing pipeline used in a recent application of a multilayer machine learning classifier to functional connectivity and coarse-scale cortical pattern analysis (McNorgan et al., 2020). Functional images were co-registered with the 3D anatomical surface generated by FreeSurfer (Version 6.0) for each participant and mapped onto a common structural template for group analysis using isomorphic 2 mm voxels. Functional data were preprocessed using FS-FAST interoperating with FSL (Version 5.0) to apply motion-correction, slice-time correction and spatial smoothing using a 4 mm Gaussian kernel. Temporal signal filtering of functional data was applied only through regression of linear trends, white matter and CSF signal, and motion parameters, however, frequency-based filtering was not applied. Functional data were mapped to FreeSurfer’s template surface space for cortical regions, and then to the MNI305 3D template space for subcortical regions.

### General Linear Model Analysis and Functional Region of Interest Generation

A general linear model analysis (GLMA) was performed in FreeSurfer’s template surface space and the MNI305 3D space at the participant level using an event-related design with the go and no-go trials included as conditions of interest (“task”) and participant motion parameters as regressors of non-interest, modeled using the SPM canonical hemodynamic response function to generate a contrast map for all task activity vs. an implicit rest baseline. This produced a functionally defined mask of cortical and subcortical regions with high signal-to-noise ratio, and we note that it included regions that were activated and deactivated relative to rest. Group-level contrasts were thresholded with a voxel-wise significance level of *p* = 0.001 and a Monte Carlo permutation simulation applied a cluster-size corrected significance level of *p* = 0.05. These whole-brain significance thresholds are commonly applied to GLMA contrasts for identifying regions showing group- or condition-differences, including previous studies of ADHD using the IGT and the go/no-go task (e.g., Suskauer et al., 2008; Yang et al., 2019).

**FIGURE 1:**
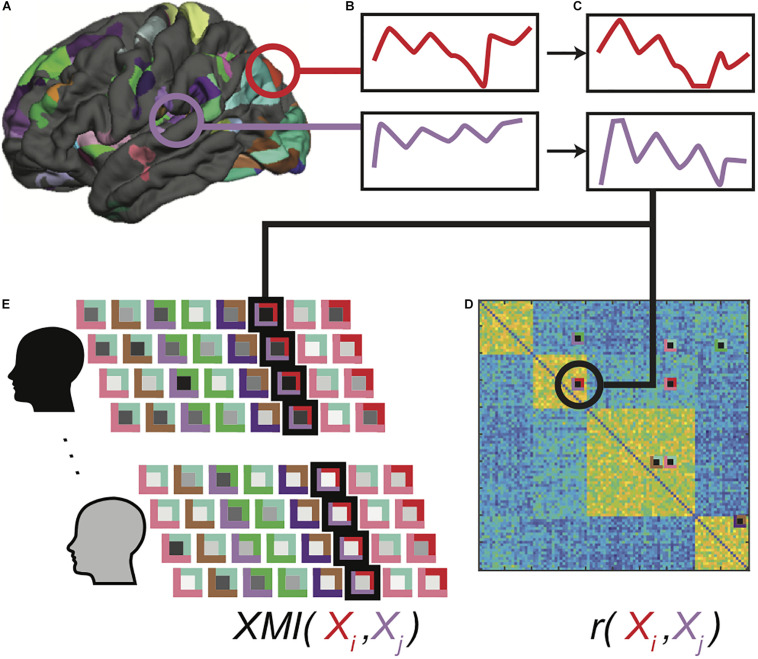
Functional data processing pipeline. Clusters showing significant task-related activation or deactivation relative to rest are sub-parcellated **(A)**, and mean BOLD time series for voxels in each region is computed **(B)**. Extracted time series are detrended, normalized and outlier values are clipped **(C)**. Pearson correlations are computed between all-time series pairs for each functional run for each participant, and region pairs with significantly correlated time series in at least 30% of all correlation matrices were identified as connections of interest **(D)**. Cross-mutual information (XMI) was calculated between the time series for each pair of regions in a connection of interest, to create a vector of XMI-based connectivity values for each functional run for each participant **(E)**. These vectors were subsequently tagged with clinical diagnosis and IGT performance classifications for the associated participants.

Large cortical patches are unlikely to be homogenously organized, and so significant group-level clusters were mapped to surface space, which the FreeSurfer mris_divide_parcellation utility algorithmically subdivided into 302 (115 left, 144 right, 42 subcortical) regions of interest (ROI) of comparable size to the Lausanne parcellation ROIs. The algorithm subdivides the vertices within each ROI perpendicular to its longest axis so that all subdivisions have roughly equal number of vertices and cover up to a designated surface area (400 mm^2^ in our study). This approach has been used in previous studies of functional connectivity in surface space (Hagmann et al., 2008; Honey et al., 2009; Hagmann et al., 2010; McNorgan and Joanisse, 2014; McNorgan et al., 2020; Figure 1A).

### Functional Connectivity and Pattern Generation

#### Correlation-Based Initial Feature Selection

As indicated earlier, functional connectivity corresponds to the temporal coherence between two brain regions, and is typically computed using the Pearson correlation between activation time series. Among *n* brain regions, we may compute *n*(*n*−1)/2 pairwise correlations, and this exponential relationship complicates the analysis and interpretation of functional connectivity: Superfluous predictors among a large number of functional connections may lead to models that overfit the training data and fail to generalize (Hawkins, 2004; Castellanos and Aoki, 2016), and it is challenging to summarize and construct a theoretical synthesis of thousands of functional connections. For these reasons, neuroimaging studies often restrict analyses to a subset of ROIs, or as appropriate for a connectivity study, a set of connections of interest (COIs). A common method of identifying meaningful functional connections within an adjacency matrix is to apply a statistical significance threshold (e.g., Tomasi and Volkow, 2011; Zeng et al., 2014). As will be explained shortly, cross-mutual information measures of dependency have several features that recommend them for use in exploration of functional connectivity. Unfortunately, in the absence of a parametric significance test for these values, they do not readily lend themselves to significance-based thresholding. For this reason, we used conventional linear correlations as an initial first pass filter in our feature selection.

Mean BOLD time series were computed across all voxels in each ROI (Figure 1B). Pairwise Pearson correlations between detrended and normalized regional time series vectors (Figure 1C) were calculated between all ROIs for each of the 4 runs, eliminating the redundant bottom triangle of the symmetric correlation matrix. The top 5% of the correlation values in at least 30% of all correlation matrices was used as a selection filter to ensure that the analyses included functional connections between brain regions that were strongly correlated in some—but not necessarily all—functional runs (Figure 1D). This selection criterion did not guarantee that the selected functional connections were strong across all individuals, or indeed, even among all functional runs for any single individual. This was by design, because a selection including only uniformly strong functional connections precludes group-related differences. Rather, this approach ensured that functional connectivity patterns varied from one another without introducing any statistical bias into the patterns associated with any classification. Moreover, because each selected functional connection was free to vary among runs within each participant, this approach prevented the classifiers from relying on idiosyncratic patterns associated with specific individuals, promoting generalizability.

#### Pattern Generation From Cross-Mutual Information Functional Connectivity

Though we used the selection filter to identify an initial set of COIs, connection strength was estimated as the cross-mutual information (XMI) (Abarbanel and Gollub, 1996) between ROI time series vectors because mutual information is more sensitive to the general dependency between two variables, which may or may not be linear (Li, 1990), is more robust to non-stationary processes commonly found in neural time series (Wollstadt et al., 2014), and may be more sensitive to synchronization in noisy systems (Paluš, 1997). Finally, mutual information values are always positive, which prevents anticorrelated regions from complicating the construction of input patterns.

The average cross mutual-information statistic is defined as

(1) $$M=\sum_{k=1}^{N_{c}}\sum_{l=1}^{N_{r}}P\,\left(k,l\right)\,\log\left[\frac{P\,\left(k,l\right)}{P\,\left(k\right)\,P\,\left(l\right)}\right]$$

Where P refers to probabilities greater than 0 on a 2-dimensional probability density: *P(k)* depicts the probability of the first variable, and *P(l)* the second variable. *P(k, l)* is the joint probability within a particular bin or range of values on the *X* and *Y* axes. *Nc* is the number of columns, representing separate bins or values across which the histogram or density function was computed for the *X* variable. *Nr* is the number of rows, representing the separate bins or values over which the histogram or density was computed for the *Y* variable. It is typically normalized by log(*Total Bins*), its maximum value for a given number of bins (i.e., *Nc* × *Nr*). While the mutual information statistic is able to capture a linear dependence between to variables, it also captures any general dependence between them. For this project, *M* was computed from the output of a two-dimensional Fast-Fourier Transform Gaussian-kernel density function. Previous work (McNorgan and Joanisse, 2014) found functional connectivity values to be normally distributed, and so the number of bins used in the XMI calculation was determined by Scott’s formula (Scott, 1979):

(2) $$T\,o\,t\,a\,l\,B\,i\,n\,s=\frac{\max X-\min X}{3.5\cdot s_{X}\cdot n^{-1/3}}$$

where *s*_*X*_ is the standard deviation of *X* and *n* is the number of values.

The XMI values were written to a connectivity vector (Figure 1E) and tagged with the clinical diagnosis (ADHD or Control) and median-split IGT score (high or low) for that participant. The classifier training data thus contained 80 participants × 4 runs = 320 tagged connectivity vectors. This dataset was augmented during training through application of feature dropout (Shorten and Khoshgoftaar, 2019), in which input features from each input pattern were set to zero with a probability of 0.4. Dropout thus simultaneously minimizes the influence of unreliably predictive features and introduces random distortions to the training patterns so that unique input patterns are presented over a large number of training events.

### Classifier Training

**FIGURE 2:**
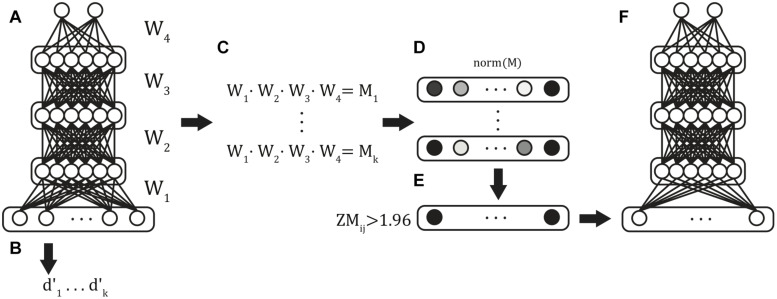
Classifier training procedure. Six batches of stratified 5-fold cross-validated models were trained from the full input feature set **(A)**, and validation-set performance was obtained for each of the resulting models **(B)**. The weights between each of the layers were extracted and matrix multiplication was used to compute the summed path weight from each feature to the classifier units **(C)**. Mean path weights were normalized **(D)**, and those features in the 5% tails were identified **(E)** and used as features in the reduced feature set model **(F)**.

Multilayer feedforward classifiers were trained using stratified k-folds cross-validation, a commonly used validation approach that ensured generalizability of model results (Figure 2A). The technique partitions the data set into training and test partitions once for each k-fold. Within each fold, the proportion of examples of each classifier category were matched in the training and test partitions. Across folds, the test folds are non-overlapping, such that all samples appear in exactly one validation set across all folds. This technique ensured that the classification accuracy reported across the entire simulation reflects the model’s ability to correctly classify all of the available data while simultaneously preventing the model from being exposed to the validation set data during training. We used fivefold cross-validation, with each fold generating one trained model and this procedure was repeated six times to produce 6 batches of 5 models (30 models total) to generate distributional statistics of model performance.

Because there were more ADHD than Control participants, we avoided biasing classification decisions by equating the group sizes through under-sampling, including the four connectivity vectors for all 25 control participants and an equal number (100 total) of randomly selected connectivity vectors for ADHD participants, fixing chance classifier accuracy to 0.5. Each batch of models used a different random subset of the ADHD patterns.

Within multilayer models, there are multiple paths through successive hidden layers between each predictor variable and classification node. A variable’s influence on the classification is thus computed by summing weights over all possible paths through multiplication of the weight matrices (Figure 2C). Classifier units imply an *activation function* that transforms the summed input. We used the logistic sigmoid activation function (Eq. 3):

(3) $$f\,\left(i\,n\,p\,u\,t\right)=\frac{1}{1+e^{-x}}$$

This function scales input to the unit interval {0,1}, so that as the summed input approaches ± ∞, the output value approaches 0 or 1. Understanding this property is critical for interpreting the network weight structure, because strong negative weights are associated with the output class of 0, and strong positive weights are associated with the output class of 1. The classifier models can be thus seen as an extension of a conventional binomial logistic regression classifier to include a series of hidden layers, described below.

**FIGURE 3:**
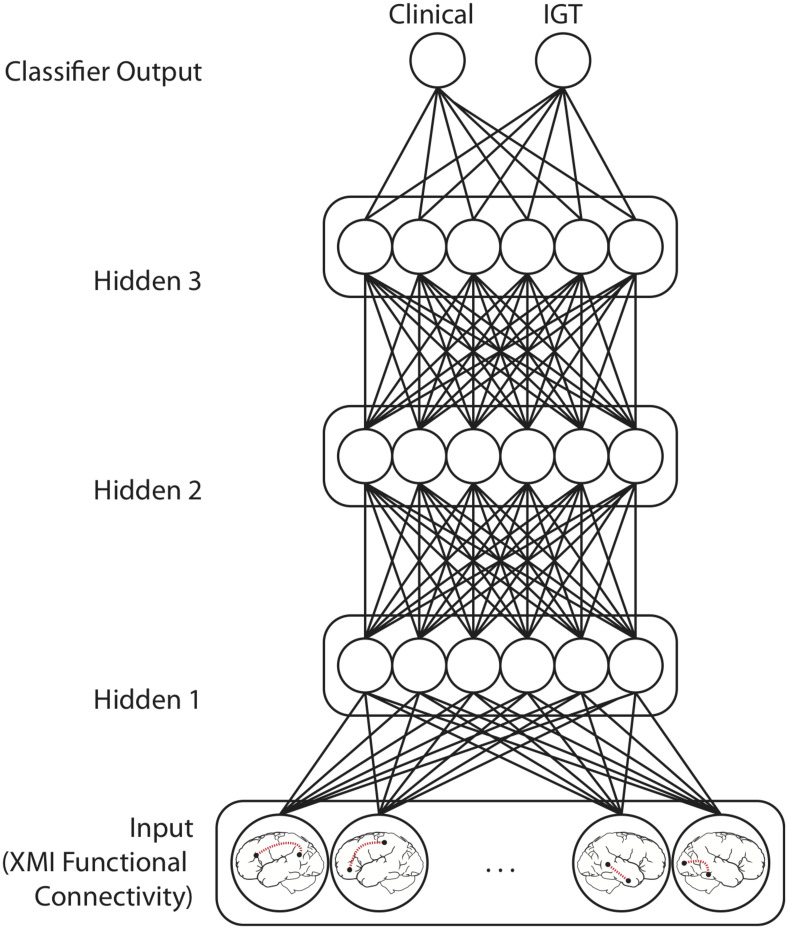
Model Architecture for networks performing mutually constrained classification. Selected functional connections within task-defined network regions were input patterns from which Clinical diagnosis (ADHD vs. Control) and IGT performance (high vs. low) was predicted. Model training iteratively adjusts the weights between the Input layer features the three Hidden layers, and the Classifier output units to minimize squared error between the target output values (0 or 1) and the classifier output values predicted for each functional connectivity pattern. Models performing only Clinical or IGT classification had only one output unit but were otherwise architecturally identical.

Classifier models were implemented in *TensorFlow* (Version 1.10)^1^. Input values fed forward through a sequence of four densely connected hidden layers, each containing 12 rectified linear units (Figure 3). Batch normalization was applied at each hidden layer (Ioffe and Szegedy, 2015). These activations fed forward to a two-unit classifier layer which simultaneously classified patterns with respect to both Clinical Diagnosis and IGT performance. This model architecture was informed by previously published applications of multilayer feedforward classifiers to neuroimaging data (Liu et al., 2018; McNorgan et al., 2020). The training set was balanced with respect to both classifications and the categories were orthogonal (i.e., knowing one classification was uninformative for the other). These models therefore identify functional connections that are predictive of both Diagnosis and IGT performance. Real-numbered output values are assigned to the category codes closest in value (e.g., an output less than 0.5 was treated as a categorization of “0”).

#### Reduced Models

Overfitting is a measurable empirical phenomenon closely related to generalizability. Prediction or classification error in a statistical or machine learning model is quantifiable by a difference metric, such as the summed squared error (e.g., in regression models) or cross-entropy (e.g., in classifier models) (Kline and Berardi, 2005). A statistical or machine learning model is said to have overfit if the error metric is small when the model is applied to the training data but large when it is applied to a novel cross-validation data set. Such a model would therefore not accurately predict outcomes for a random sample drawn from the population, limiting its utility for informing generalizable theories. It is not uncommon for machine learning models to achieve perfect performance for the training data, and thus some discrepancy between training and validation set performance is expected. However, overfitting is continuous and measurable, and so one approach is to measure validation set accuracy over a series of replications using randomized models to obtain distributional statistics for validation set accuracy.

The feature selection step described above generated input feature vectors containing 2265 features. For simple models such as standard two-layer support vector machines (SVMs) or logistic regression models, in which each input feature directly influences classification, high-dimensional input patterns might raise concerns of the potential for overfitting the training data in two related senses (Hawkins, 2004): First, superfluous input features provide additional opportunities for idiosyncratically predictive features to inflate model performance. Second, superfluous predictors lead to unparsimonious models that do little to advance theory. An advantage of our multilayer model architecture is that hidden layers introduce a bottleneck into the transmission of the input pattern to the classifier units. In addition to the feature selection step prior to training pattern generation, this architectural feature implements a feature reduction step by requiring the network to create a 12-dimensional non-linear independent components analysis (ICA) recoding of the input pattern (DeMers and Cottrell, 1993; Lotlikar and Kothari, 2000; Hyvärinen and Bingham, 2003). By implementing the feature reduction step within the model architecture, rather than as a preprocessing step, the contributions of individual features from the intact dataset may be evaluated. Moreover, because the ICA is trained by the same error signals that drive the classification boundaries, the discovered components should be optimized with respect to the classification decision. Though regularization techniques during training and ICA reduction improve generalizability to novel data, it remains challenging to meaningfully discuss more than a handful of individual functional connections. We thus further reduced the feature set, appealing to the logic of backward step-wise regression.

The feature selection procedure leaks information about the most informative features between models using the full set of features and those using a reduced feature set, however, this is not problematic for two reasons: First, feature selection was intended to facilitate interpretation, rather than improve accuracy; the survival and subsequent inclusion of predictor x*_*i*_* in the reduced model generation is analogous to the survival of predictor *x*_*i*_ into the *n* + 1th step in a backward stepwise multiple regression. Second, each of the 6 model batches are independent, precluding information leakage *between* batches. The analyses that follow aggregate results across all model batches, permitting measures of predictive reliability for each functional connection, and more importantly, the evaluation of a model comprising the most informative features independently identified by each batch of models.

We first evaluated the performance of 30 trained models on the full input vectors (Figure 2B). Next, after normalizing the summed path weights (Figure 2D), we identified the functional connections with path weights to the ADHD classification unit in the ± 0.025 tails of the distribution of weights in all models (Figure 2E). This selection further reduced our input patterns to include only those XMI functional connectivity values that were most diagnostic of ADHD classification across all random models. Finally, we repeated the above fivefold cross-validation procedure, training on the reduced input space to generate six fivefold batches (30 models total) of Reduced-Feature Models (Figure 2F). We report the classification performance of the Reduced Models below.

### Model Evaluation

Validation set accuracy was used to evaluate the efficacy of functional connectivity in the classification decisions on which the models were trained. The relative influences of individual functional connections on classification decisions were evaluated by computing the summed path weights from each input unit (each encoding the functional connectivity between a pair of brain regions) to each of the classifier units. In addition to parametric assessments of predictive functional connections under a normal distribution, a non-parametric assessment was performed by comparing classification performance for networks with weights from influential units selectively removed against performance of networks with an equivalent number of randomly selected weights removed. Classification performance should be more impacted by the selective removal of the input features we identified as highly diagnostic.

## Results

We report several measures of model performance, computed across all fivefold for each of the 6 batches (*n* = 30) of models. These measures include mean classification accuracy (*M*), hit rate, false alarm rates, and d-prime recorded for both clinical diagnosis and IGT classifications. Because ADHD was mapped to zero, a true positive was a correct classification of Control to category 1, and a true negative was a correct classification of ADHD to category 0. Signal detection theory measures of model performance were thus defined by formulas 4, 5, and 6 for hit rate (HIT), false alarm rate (FAR), and d-prime (d′):

(4) $$H\,I\,T=\frac{t\,r\,u\,e\,p\,o\,s\,i\,t\,i\,v\,e}{t\,r\,u\,e\,p\,o\,s\,i\,t\,i\,v\,e+f\,a\,l\,s\,e\,n\,e\,g\,a\,t\,i\,v\,e}$$

(5) $$F\,A\,R=\frac{f\,a\,l\,s\,e\,n\,e\,g\,a\,t\,i\,v\,e}{f\,a\,l\,s\,e\,n\,e\,g\,a\,t\,i\,v\,e+t\,r\,u\,e\,n\,e\,g\,a\,t\,i\,v\,e}$$

(6) $$d^{\prime }=z\,\left(F\,A\,R\right)-z\,\left(H\,I\,T\right)$$

Where *z*(*X*) is the Z score corresponding to the right-tail *p*-values associated with proportion *X*. Single-sample right-tailed *t*-tests against chance (0.5) were Bonferroni-Holm corrected for multiple comparisons, which was chosen because it is the most conservative correction. Both full and reduced feature set models demonstrated very high classification performance on clinical diagnosis and IGT performance classifications, explicitly linking IGT performance with ADHD through a shared connectomic fingerprint.

Because model weights are shared between output categories, classifier training for both categories constrains the solution space to the set of functional connections that are *optimally* diagnostic for *both* types of classification (McNorgan et al., 2020). Both classifications were at well over chance accuracy (*M*_*Clinical*_ = 0.91, *SD* = 0.07, *t*(29) = 32.78, *p* < 0.00001; *M*_*IGT*_ = 0.91, *SD* = 0.06, *t*(29) = 34.10, *p* < 0.00001). The HIT, FAR and d-prime scores indicate the models achieved high accuracy through both high sensitivity and high specificity (*HIT*_*Clinical*_ = 0.86, *FAR*_*Clinical*_ = 0.04, *d′_*Clinical*_* = 2.90; *HIT*_*IGT*_ = 0.83, *FAR*_*IGT*_ = 0.02, *d′_*IGT*_* = 3.03). Finally, we observe that these values represent per-run performance (i.e., for connectivity obtained from just one of four runs). Thus, if classification used the modal classification of all four connectivity matrices, participant-level accuracy rises to 0.991, or 99%. The high accuracy suggests a relatively robust consistency in the functional connections that distinguish the two groups.

We may compare our multilayer feedforward classifier architecture with classification performance for a more conventional linear SVM classifier, to appreciate the benefit of the embedded ICA enabled by the hidden layer transformations on the input pattern. With only a single classification hyperplane, series of SVM classifiers on random balanced subsets of the training data demonstrated worse validation set classification accuracy for both clinical diagnosis (*M*_*Clinical*_ = 0.58, *SD* = 0.05) and IGT performance (*M*_*IGT*_ = 0.86, *SD* = 0.07). The classification performance is attributable to poor sensitivity for clinical diagnosis (*HIT*_*Clinical*_ = 0.16, *FAR*_*Clinical*_ = 0.00). We will clarify that the FAR is reported in the context of a “hit” mapped to the Control category: though the classes were balanced, the SVM models classified 93% of all patterns as ADHD, showing clearly biased classification decisions across all randomized simulations. The SVM FAR is thus indicative of a reluctance to assign any pattern to the Control diagnosis, rather than of very high specificity. This difference reinforces the importance of feature reduction for mitigating overfitting, and of non-linear relationships in the classification decisions—particularly for clinical diagnosis. We also observe that the linear SVM classifier cannot make simultaneous classification decisions of two orthogonal categories. These classifiers were trained to make clinical and IGT classifications independently, precluding the possibility of identifying interactions among predictive features for the two classifications.

### Diagnostic Functional Connectivity

The ADHD classification was mapped to a Clinical output value of zero, and thus strong negative weights to the Clinical classifier unit was predictive of an ADHD diagnosis (by implication, weak positive connectivity was therefore predictive of an ADHD diagnosis). Low IGT performance was mapped to an IGT output value of zero. To facilitate interpretation, we normalized the summed path weights between each functional connection and classification output. Functional connections that are predictive of both Clinical and IGT classification would have high absolute value weights to both outputs. Thus, we identified functional connections with an absolute value of either normalized weight greater than | Z| = 1.65 (95th percentile), and highlight those for which the product of weights was greater than 1.65^2^, indicating weights in the extreme tails for both classifications. These *highly predictive* functional connections for which strong connectivity is most diagnostic of an ADHD diagnosis are reported in Table 1, and those for which strong connectivity predicts a Control diagnosis—and therefore weak connectivity is diagnostic of an ADHD diagnosis—are reported in Table 2. In these tables, we report the normalized mean model path weights between each predictive functional connection and the classifier units for Clinical and IGT classifications. These weights are sorted by the product of their absolute values. Highly predictive functional connections are denoted with an asterisk.

**TABLE 1:** Normalized mutual constraint model weights for strong functional connections predictive of ADHD diagnosis.

**IGT class**	**Clinical**	**IGT**	**Label**	**X**	**Y**	**Z**	**Label**	**X**	**Y**	**Z**	**Product**
High IGT	–5.32	1.57	Occipital Mid. L	–39	–74	32	Occipital Mid. L	–39	–70	38	−8.33*
	–2.61	2.66	Temporal. Mid R	48	–55	17	Temporal Mid. R	49	–56	11	−6.94*
	–1.98	2.85	Cingulum Post. L	–6	–30	29	Cingulum Mid. L	–6	–27	30	−5.64*
	–2.63	1.47	Rolandic Oper. R	54	–18	19	Insula R	34	–16	16	−3.88*
	–1.82	1.54	Precentral R	31	–25	56	Postcentral R	27	–42	59	−2.80*
	–1.04	2.57	Fusiform R	29	–83	–3	Calcarine R	22	–86	1	–2.67
	–1.73	1.45	Occipital Mid. L	–17	–93	6	Occipital Mid. L	–24	–87	6	–2.50
	–2.50	0.94	Temporal Sup. R	51	–35	15	Temporal Sup. R	40	–27	10	–2.35
	–2.40	0.98	Temporal Mid. R	54	–42	–1	Temporal Mid. R	49	–56	11	–2.34
	–0.51	4.28	Calcarine L	–7	–85	13	Calcarine R	9	–72	14	–2.20
	–2.22	0.97	Insula L	–41	15	7	Insula L	–35	18	0	–2.15
	–2.69	0.76	Angular R	36	–55	42	SupraMarginal R	49	–40	41	–2.06
	–0.94	2.16	Calcarine L	–7	–85	13	Calcarine L	–8	–79	12	–2.03
	–5.02	0.37	Occipital Mid. R	29	–65	26	Temporal Mid. R	49	–56	11	–1.83
	–0.57	2.97	Temporal Sup. R	51	–35	15	SupraMarginal R	43	–28	34	–1.69
	–0.49	3.27	Putamen R	36	6	0	Insula R	35	15	–3	–1.61
	–0.48	2.85	Calcarine R	9	–83	13	Fusiform R	30	–69	–2	–1.38
	–2.84	0.30	Temporal Inf. R	47	–51	–14	Occipital Inf. R	39	–66	–14	–0.85
	–3.77	0.15	SupraMarginal R	58	–41	30	Angular R	53	–49	32	–0.56
	–0.16	3.24	Postcentral R	55	–10	33	Precentral R	42	–10	45	–0.53
Low IGT	–3.73	–2.27	Fusiform L	–36	–66	–10	Occipital Inf. R	44	–75	–11	8.49*
	–2.72	–1.39	Fusiform L	–42	–58	–13	Occipital Inf. L	–30	–82	–8	3.78*
	–3.51	–1.03	Lingual R	16	–53	–3	Lingual R	18	–43	–5	3.62*
	–3.17	–1.00	Occipital Sup. L	–10	–97	7	Calcarine L	–19	–78	6	3.17*
	–2.23	–1.37	Paracentral Lobule L	–14	–34	66	Postcentral L	–24	–36	57	3.06*
	–2.31	–1.12	Caudate L	–13	19	8	Caudate R	13	19	8	2.60
	–2.81	–0.92	Fusiform L	–33	–73	–15	Lingual L	–11	–77	–5	2.58
	–1.09	–2.15	Precuneus R	11	–49	56	Postcentral R	13	–36	64	2.35
	–2.23	–1.05	Lingual L	–14	–54	–4	Lingual R	16	–53	–3	2.33
	–0.81	–2.34	Calcarine L	–8	–79	12	Calcarine R	9	–83	13	1.89
	–0.34	–2.76	Paracentral Lobule L	–14	–26	66	Insula L	–28	28	3	0.94
	–0.28	–2.92	Fusiform R	30	–69	–2	Calcarine R	25	–55	11	0.82
	–0.23	–3.44	Lingual L	–11	–77	–5	Lingual R	17	–62	–6	0.79
	–0.22	–2.97	Occipital Mid. L	–24	–87	6	Lingual L	–22	–76	–8	0.66

**TABLE 2:** Normalized mutual constraint model weights for strong functional connections predictive of Control diagnosis.

**IGT Class**	**Clinical**	**IGT**	**Label**	***X***	***Y***	***Z***	**Label**	***X***	***Y***	***Z***	**Product**
High IGT	4.28	2.77	Cingulum Ant. L	–12	44	2	Frontal Med. Orb R	11	51	–3	11.82*
	2.96	2.67	Fusiform L	–33	–73	–15	Occipital Inf. L	–16	–92	–9	7.90*
	3.29	2.35	Occipital Inf. R	39	–66	–14	Fusiform R	38	–53	–18	7.73*
	2.54	1.95	Calcarine L	–7	–85	13	Lingual R	8	–69	2	4.94*
	1.53	3.10	Precentral R	26	–25	52	Rolandic Oper. R	39	–30	20	4.75*
	3.92	1.19	Occipital Inf. L	–16	–92	–9	Lingual R	26	–84	–9	4.66*
	1.50	3.06	Postcentral L	–26	–34	64	Postcentral L	–24	–36	57	4.60*
	1.96	2.06	Temporal Inf. R	47	–51	–14	Fusiform R	38	–53	–18	4.03*
	2.50	1.48	Postcentral L	–43	–17	36	Postcentral L	–59	–12	30	3.71*
	1.61	2.29	Calcarine L	–17	–73	6	Calcarine R	21	–67	9	3.69*
	2.31	1.50	Angular L	–36	–58	40	Angular R	41	–59	29	3.47*
	2.32	1.31	Insula R	34	17	10	Insula R	35	15	–3	3.03*
	1.19	2.42	Fusiform L	–26	–76	–6	Lingual R	10	–91	–7	2.88*
	1.60	1.77	Precentral L	–38	–8	50	Precentral L	–40	–2	46	2.83*
	4.12	0.67	Frontal Inf. Oper. R	49	13	15	Frontal Inf. Oper. R	52	13	9	2.76*
	2.40	1.14	Occipital Sup. L	–20	–80	38	Parietal Sup. L	–17	–70	44	2.75*
	1.29	2.11	Frontal Sup. Medial L	–19	39	17	Frontal Med. Orb R	11	51	–3	2.74*
	1.64	1.61	Lingual L	–21	–67	4	Calcarine R	9	–72	14	2.65
	1.70	1.51	Calcarine R	9	–72	14	Lingual R	8	–69	2	2.58
	2.05	1.15	Cuneus L	–7	–63	24	Precuneus L	–11	–57	13	2.37
	2.14	1.09	Cingulum Mid. L	–10	15	44	Supp Motor Area L	–11	7	49	2.34
	2.77	0.79	Cingulum Ant. L	–12	44	2	Frontal Sup. Medial L	–19	39	17	2.19
	0.59	3.11	Calcarine L	–7	–85	13	Calcarine R	15	–71	6	1.83
	0.59	2.79	Precuneus L	–12	–47	60	Paracentral Lobule R	14	–41	53	1.65
	2.59	0.57	Postcentral R	38	–21	47	Postcentral R	38	–17	37	1.49
	3.01	0.24	Temporal Sup. L	–47	–28	4	Temporal Sup. L	–50	–18	3	0.71
	0.05	3.65	Amygdala L	–32	–1	–16	Amygdala R	32	–1	–16	0.17
	3.35	0.03	Temporal Mid. R	56	–46	1	Temporal Mid. R	56	–34	–8	0.10
Low IGT	3.86	–1.45	Frontal Inf. Oper. R	43	10	20	Frontal Inf. Oper. R	46	17	19	−5.61*
	1.81	–2.85	Cingulum Post. L	–14	–93	–16	Cingulum Post. R	14	–93	–16	−5.16*
	3.52	–1.28	Frontal Med. Orb R	11	51	–3	Frontal Med. Orb R	10	49	–3	−4.49*
	2.03	–1.66	Lingual L	–18	–82	–8	Lingual R	17	–62	–6	−3.36*
	1.67	–1.72	Occipital Mid. R	29	–65	26	Caudate R	13	19	2	−2.88*
	1.39	–2.03	Rolandic Oper. R	39	–30	20	Rolandic Oper. R	45	–13	18	−2.82*
	1.63	–1.71	Non-Task	0	0	0	L Posterior Cingulate	–14	–93	–16	−2.78*
	2.03	–1.19	Calcarine R	9	–83	13	Lingual R	8	–69	2	–2.41
	2.10	–1.03	Precuneus R	11	–49	56	Precentral R	26	–25	52	–2.16
	3.47	–0.61	Angular R	30	–60	44	Occipital Sup. R	31	–64	42	–2.13
	0.60	–3.41	Occipital Inf. R	44	–75	–11	Occipital Inf. R	36	–68	–9	–2.06
	0.20	–3.22	Fusiform L	–36	–66	–10	Calcarine L	–7	–85	13	–0.66
	3.31	–0.18	Occipital Inf. R	39	–66	–14	Occipital Inf. R	33	–78	–6	–0.59
	0.16	–3.57	Lingual R	26	–84	–9	Fusiform R	29	–83	–3	–0.55
	0.02	–3.63	Temporal Mid. R	48	–55	4	Fusiform R	29	–83	–3	–0.07
	0.01	–3.21	Non-Task	0	0	0	Amygdala R	32	–1	–16	–0.04

Though our *highly predictive* connections were identified using conventional parametric thresholds for determining significance, the assumptions underlying conventional parametric analyses may not be justified for direct parametric analyses of model weights (Luengo et al., 2009). Consequently, it does not necessarily follow that the highly predictive connections are significantly better for prediction than other functional connections within the task-defined network. A permutation test contrasted the predictive accuracy for a series of random networks trained using only the subset of highly predictive connections as inputs and an equal number of networks trained using an equal number of randomly selected functional connections. Mean test-set classification accuracy was computed for 10-fold of 10 sets of (highly predictive and random-feature) models trained using stratified cross-validation. Independent-samples *t*-tests found that the highly predictive functional connections predicted Clinical diagnosis with higher accuracy (*M* = 0.76, *SD* = 0.01) than did random feature networks (*M* = 0.70, *SD* = 0.02), *t*(18) = 8.10, *p* < 10^–6^. The highly predictive functional connections also predicted IGT performance with higher accuracy (*M* = 0.72, *SD* = 0.02) than did random feature networks (*M* = 0.69, *SD* = 0.01), *t*(18) = 4.13, *p* = 0.0003. Thus, the highly predictive functional connections were significantly better predictors for both classifications than were a comparable set of randomly selected functional connections from the reduced feature set.

**FIGURE 4:**
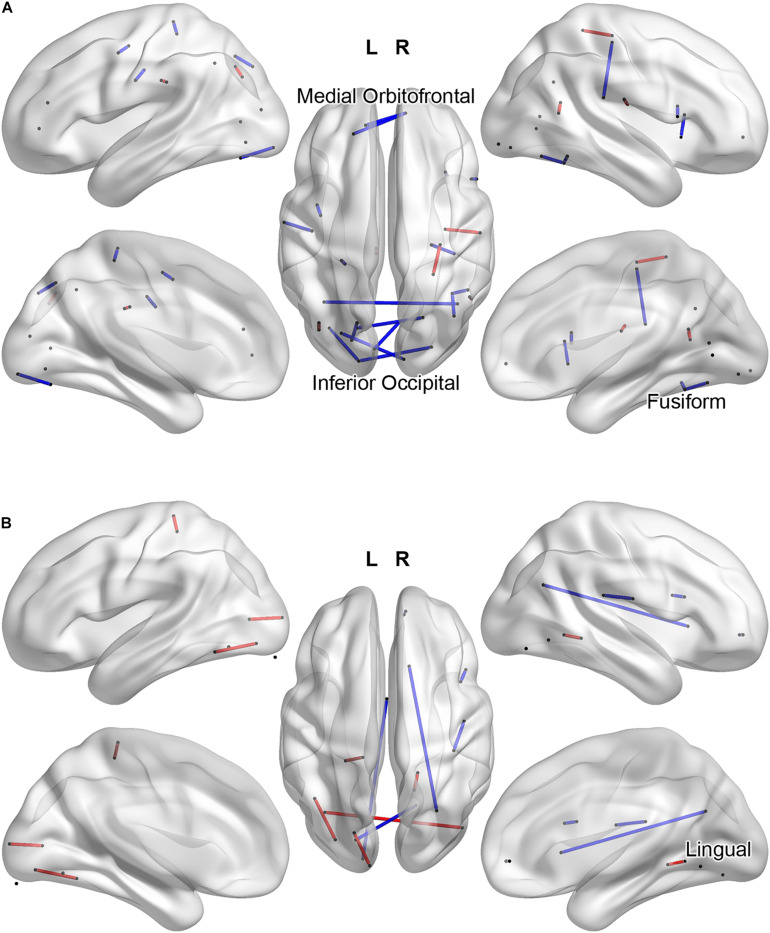
Task-related functional connections most predictive of Clinical Diagnosis and high IGT scores **(A)** and most predictive of Clinical Diagnosis and low IGT scores **(B)**. ADHD hypoconnectivity appears in blue; ADHD hyperconnectivity appears in red. Regions involved in multiple predictive connections are labeled.

If we interpret the functional connections listed in Table 2 as those for which low connectivity values are predictive of an ADHD diagnosis, we may instead group these weights with respect to IGT performance and compare functional connectivity profiles in terms of the hyper- and hypoconnectivity predicting an ADHD diagnosis. Figure 4A plots the functional connections predicting diagnosis and a high IGT score: ADHD hypoconnectivity appears in blue and ADHD hyperconnectivity appears in red. Network descriptions are derived from the Yeo et al. (2011) 7-network parcellation. For those with high IGT performance, models predict an ADHD diagnosis from interhemispheric hypoconnectivity between the visual and ventral attention network involving inferior occipital and fusiform cortex in posterior regions; interhemispheric hypoconnectivity within the anterior DMN, involving medial orbitofrontal cortex; and hypoconnectivity between the right motor network and the ventral attentional network involving the right supramarginal and precentral gyri. Additionally, hyperconnectivity within left ventral attention network predicts ADHD concurrent with high IGT performance. Figure 4B illustrates a different connectivity profile associated with low IGT performance concurrent with an ADHD diagnosis. This classification was associated with hyperconnectivity between the visual network and ventral attention network, involving left fusiform and bilateral inferior occipital cortex, and hypoconnectivity within the posterior DMN between lingual gyrus and posterior cingulate gyrus, and between lingual gyrus to regions outside the task-defined network.

## Discussion

This study made several novel contributions to the understanding of ADHD. First, we applied machine learning classifiers to task-related functional connectivity from the go/no-go task. The high accuracy achieved by these models further suggests XMI-based measures as useful metrics of functional connectivity. Second, ours was the first study to make multiple orthogonal classifications from whole-brain neural activations, allowing us to establish the mutual relevance of functional connectivity for IGT performance and ADHD diagnosis. Because the classifications were based on shared model parameters, our results show not only *that* IGT performance is relevant to ADHD diagnosis, but by identifying the shared informative connections, also show *why* this task is behaviorally relevant. Finally, where IGT performance is one of several diagnostic tools, it may discriminate between ADHD subtypes and inform treatment.

### Parametric Decisions

Our model architecture and training parameters were informed by previous work (Liu et al., 2018; McNorgan et al., 2020) and by iterative tuning of model hyper-parameters. Cross-validation explicitly guards against the primary concern with parametric tuning of mathematical models; namely, that model optimization comes the expense of external validity and generalizability to new data. Our modeling approach is not specific to our dataset, and may be applied to other measures or domains. Such applications may find our parametric choices to be a useful starting point, and automated tools for parametric space optimization, such as GPflowOpt (Knudde et al., 2017), which algorithmically explore the hyperparametric space, can facilitate development of optimized models without sacrificing generalizability.

Though we used conventional correlation-based measures of connectivity during feature selection, we chose to use XMI-based measures in our training patterns precisely because this measure is infrequently used in functional connectivity studies (and thus begs exploration) but also likely to be sensitive to the sorts of non-linear relationships we anticipated in a categorization problem built around non-linearly separable classes among time series that may be non-stationary (Wollstadt et al., 2014). Our results should not be construed to imply that XMI-based measures are necessarily superior to other univariate or multivariate measures, e.g., as described in Nieto-Castanon (2020). An exposition of the relative merits of alternative measures of functional connectivity is beyond the scope of this study and would require ground-truth knowledge of the connectivity in our data, but our results suggest that XMI may be worth consideration in the analysis of functional connectivity.

### Relation to Previous Work

Aligning our results with a literature that has largely focused on rs-fMRI using seed-based approaches is a challenge compounded by our identification of predictive connectivity using a joint probability distribution function over clinical diagnosis and IGT performance. Nonetheless, several functional connections from the resting state literature were also predictive in our task data. High IGT performance concurrent with ADHD was predicted by hypoconnectivity between anterior cingulate and orbitofrontal cortex, regions implicated in reward-motivation and salience attribution, respectively. Hyperconnectivity between these regions was found by Tomasi and Volkow (2012) using rs-fMRI. This apparent contradiction can be reconciled by the fact that rs-fMRI is task-negative, suggesting that an inability to appropriately engage and disengage these two systems is a defining feature of some individuals with ADHD. Predictive hypoconnectivity between visual and ventral attention network we observed has also been found using rs-fMRI (Ergül et al., 2019) in adults with social anxiety disorder with comorbid ADHD, but not others.

Few studies have combined fMRI and machine learning to the exploration ADHD. SVM classification of DMN connectivity among children, adults with ADHD and age-matched controls found that ADHD is associated with delayed maturation of this brain circuit (Sato et al., 2012). The ADHD-200 Global Competition saw several groups (Cheng et al., 2012; Colby et al., 2012; Dey et al., 2012) apply SVM linear classifiers to functional connectivity measures derived from a multi-site rs-fMRI dataset^2^ to identify characteristic ADHD rs-fMRI connectivity profiles. Cheng and colleagues (Cheng et al., 2012) were able to classify ADHD participants with 76% accuracy, finding altered frontal and parietal connections were most diagnostic. Colby and colleagues (Colby et al., 2012) classified ADHD participants with 55% accuracy using only graph-theoretic metrics, precluding identification of diagnostic connections. Dey et al. (2012) achieved roughly 70% classification accuracy using predictive graph theoretic metrics, finding that voxel selection using a functional mask, as applied in the present study, greatly improved classification accuracy by eliminating potential sources of noise. The difference in classification performance between our study and these earlier studies suggests that, though summary metrics quantifying connectivity motifs in core functional networks are predictive of ADHD, information about specific connections provides a great deal of additional diagnostic information. Guo et al. (2020) demonstrated that SVM classifiers were able to identify ADHD male adults from rs-fMRI connectivity measured among ADHD children with 76% accuracy, after first selecting the top 2% of diagnostic features from alternative models—similar to the feature reduction step employed in the present study. The authors argue that, though the predictive features may vary somewhat across cohort, the reasonable cross-cohort performance suggests that resting state functional connectivity may be a developmentally stable biomarker of ADHD. Though no cross-cohort classification was performed in the present study, accurate discrimination of childhood ADHD diagnosis from task-dependent functional connectivity in young adults further supports functional connectivity as a developmentally stable biomarker for ADHD.

Our study design is most similar to recent studies by Wang et al. (2018) and Jung et al. (2019) that applied machine learning classifiers to whole-brain rs-fMRI functional connectivity. These studies achieved ADHD classification accuracy of 75 and 84%, respectively, and both identified bilateral visual to DMN hypoconnectivity associated with ADHD. The present study also found hypoconnectivity from the right visual network to a region within the anterior DMN was strongly predictive of ADHD, but only for those who did relatively poorly on the IGT; for those who did well on the IGT, interhemispheric hypoconnectivity *within* the visual network was predictive of ADHD, contrary to the pattern reported by Wang and colleagues. This is easily reconciled by observing that ADHD is typically associated with poor IGT performance, and thus that the parallel classifications enabled our model to categorically partition typical and atypical ADHD profiles. Both Wang and colleagues and Jung and colleagues additionally identified hyperconnectivity between several regions over several functional networks, whereas hyperconnectivity was seldom highly predictive in our fMRI-based connectivity data, found primarily within the visual network for individuals showing the characteristic poor IGT performance profile. Combined with our results, this pattern suggests that resting state hyperconnectivity but task-related hypoconnectivity may be characteristic of ADHD, indicating a general difficulty in task-appropriate engagement and disengagement of multiple functional networks, but that individuals with ADHD that perform atypically well on the IGT demonstrate a different connectivity profile within the visual processing network. Whether these individuals might constitute a distinct subgroup is a matter for further clinical investigation, but these results suggest that some behavioral profiles among those diagnosed with ADHD may respond differently to treatments that target different attentional systems.

We achieved superior classification accuracy compared to these earlier studies, and ascribe this improvement to several factors: First, as demonstrated by Dey et al. (2012), the restriction of our analyses to task-relevant voxels using a functional mask reduced noise among the classifier features, and likely optimized model performance. Second, and relatedly, there is a strong theoretical connection between the inhibitory processes implied by the go/no-go fMRI task and both the IGT and ADHD. By focusing on the neural substrates supporting these processes, our analyses may have been more likely to identify mutually predictive connectivity patterns. Third, our XMI measure may be more sensitive to non-linear coactivation relationships. Finally, multilayer feedforward models have the computational flexibility to encode conditional relationships that linear SVM classifiers cannot, by internally constructing a lower-dimensional representation of the input data that is optimized with respect to the classification decision. With multiple subtypes, it is widely accepted that ADHD is not a monolithic disorder, and different network dynamics may underlie different subtypes. We have demonstrated here the benefits of the increased flexibility and sensitivity afforded by multilayer networks over their simpler counterparts, and recommend their application for answering questions that cannot be addressed by more conventional approaches, such as SVMs and logistic regression.

## Conclusion

The high classification accuracy, diagnosticity and specificity of our multilayer classifier models show ADHD is reliably predicted by task-based functional connectivity. Simultaneous prediction of IGT performance suggests that diagnosticity of the IGT is attributable to its shared reliance on clinically diagnostic functional connections. Our improved accuracy over earlier studies highlights the importance of connections involving task-positive regions and of non-linear relationships in understanding neural processing dynamics. Our multiple constraint network analysis is generalizable to other behavioral assessments and domains, and may guide development of more efficacious intervention strategies.

## Data Availability Statement

The data used in the preparation of this article were obtained from the NIH Pediatric MRI Data Repository created by the NIH MRI Study of Normal Brain Development (https://nda.nih.gov/). This is a multisite, longitudinal study of typically developing children from ages newborn through young adulthood conducted by the Brain Development Cooperative Group and supported by the National Institute of Child Health and Human Development, the National Institute on Drug Abuse, the National Institute of Mental Health, and the National Institute of Neurological Disorders and Stroke (Contract Nos. N01-HD02-3343, N01-MH9-0002, and N01-NS-9-2314, -2315, -2316, -2317, -2319 and -2320). A listing of the participating sites and a complete listing of the study investigators can be found at http://pediatricmri.nih.gov/nihpd/info/participating_centers.html.

## Author Contributions

CM conceived and supervised data analysis, coding, and computational tasks, and took primary responsibility for writing the manuscript with input from CJ and JH. CJ worked out the technical details and implemented the computational model with input from CM. DH audited and performed a naive validation of the computational model and optimized computational model code for Python 3.x to improve readability and maintainability. JH provided the initial conceptual focus and aided in writing the manuscript. All authors contributed to the article and approved the submitted version.

## Conflict of Interest

The authors declare that the research was conducted in the absence of any commercial or financial relationships that could be construed as a potential conflict of interest.

## Footnotes

1 https://www.tensorflow.org

2 http://neurobureau.projects.nitrc.org
